# Research topics and trends in medical education by social network analysis

**DOI:** 10.1186/s12909-018-1323-y

**Published:** 2018-09-24

**Authors:** Young A Ji, Se Jin Nam, Hong Gee Kim, Jaeil Lee, Soo-Kyoung Lee

**Affiliations:** 1 Biomedical Knowledge Engineering Laboratory, Seoul National University, Seoul, Republic of Korea; 2 National Center of Excellence in Software, Chungnam National University, Daejeon, Republic of Korea; 3 Center for Innovative in Dental Education, Seoul National University, Seoul, Republic of Korea; 4 College of Nursing, Keimyung University, 1095 Dalgubeol-daero, Dalseo-Gu, Daegu, 42601 Republic of Korea

**Keywords:** Research trends, Research topics, Medical education, Social network analysis, Complex systems theory

## Abstract

**Background:**

As studies analyzing the networks and relational structures of research topics in academic fields emerge, studies that apply methods of network and relationship analysis, such as social network analysis (SNA), are drawing more attention. The purpose of this study is to explore the interaction of medical education subjects in the framework of complex systems theory using SNA and to analyze the trends in medical education.

**Methods:**

The authors extracted keywords using Medical Subject Headings terms from 9,379 research articles (162,866 keywords) published in 1963–2015 in PubMed. They generated an occurrence frequency matrix, calculated relatedness using Weighted Jaccard Similarity, and analyzed and visualized the networks with Gephi software.

**Results:**

Newly emerging topics by period units were identified as historical trends, and 20 global-level topic clusters were obtained through network analysis. A time-series analysis led to the definition of five historical periods: the waking phase (1963–1975), the birth phase (1976–1990), the growth phase (1991–1996), the maturity phase (1997–2005), and the expansion phase (2006–2015).

**Conclusions:**

The study analyzed the trends in medical education research using SNA and analyzed their meaning using complex systems theory. During the 53-year period studied, medical education research has been subdivided and has expanded, improved, and changed along with shifts in society’s needs. By analyzing the trends in medical education using the conceptual framework of complex systems theory, the research team determined that medical education is forming a sense of the voluntary order within the field of medicine by interacting with social studies, philosophy, etc., and establishing legitimacy and originality.

**Electronic supplementary material:**

The online version of this article (10.1186/s12909-018-1323-y) contains supplementary material, which is available to authorized users.

## Background

An ancient scholar, Aristotle, established the basis of predicate logic, which divides knowledge into the smallest units and expresses it by linking them together (Sung-ho H: Structure and emergence analyses of knowledge network based on the social network analysis (SNA) methods: Focused on chungcheong strategic industries, unpublished) [1]. Recent studies have begun to use social network analysis as a means to analyze the trends of studies and understand the knowledge systems of each field by analyzing previously researched results. Generally, the purpose of research trend analysis in a particular academic field is to comprehend the current state of research by examining the existing results and to present future research directions [2].

Research trend analysis has been conducted for articles published in representative journals in medical education (ME), and its results serve as fundamental measures for securing academic identity [3, 4]. Analysis is being conducted from multiple angles to confirm this identity from a holistic perspective, and research methods analyzing the relationship through the application of SNA in research trend analysis are steadily increasing in the social studies field.

The study of trends in medical education analyzes the entire academic field or the subject of a particular academic field. There are studies taking quantitative approaches such as those analyzing the frequency of medical training in medical education [4–8]. Other examples include studies that a) focused on the main subjects studied in medical education by analyzing common research topics in medical education from six journals [4], b) analyzed the co-topics occurring frequently in ME articles and the differences among journals’ publication of co-topics [9], c) focused on top-cited articles identified by keyword search [10], and d) focused on network analysis of the researchers in medical education [11]. In addition, analysis of unit subjects in specific academic fields, such as an analysis of the trends in research topics including a study on the geographical distribution of researchers whose works have published in major journals of medical education [12] and a study on the social relationships of medical students and the dispersion of their attitudes [13], have been conducted steadily each year.

These studies are meaningful in that they analyze the trends of medical education subjects from a macro perspective or study specific research topics from a micro perspective, thus enabling the analysis of the trends in medical education and its knowledge system. However, this method requires the collection and analysis of vast amounts of data, demanding considerable time and manpower for interpretation. In addition, it is highly likely that researchers rely on the knowledge, experience, and insight of experts during analysis [14, 15]. In addition, the analysis has to be conducted by sorting the impactful keywords based on frequency [9, 11] or citation factors [10] or through keyword analysis by topics [4]. However, such methods are limited in their ability to identify historical changes in the relationships between specific topics. SNA is a commonly accepted method for quantitatively and visually obtaining the overall structures of network connections.

As studies analyzing the networks and relational structures of research topics in academic fields emerge, studies that apply methods of network and relationship analysis, such as SNA, are drawing more attention [16]. General methods of analyzing research trends include using co-word analysis on keywords extracted from databases [10, 11], co-citation analysis using the citation information of articles [12]. And there have also been studies on topics network analysis [13, 16–18].

SNA is an actively utilized method that recognizes and interprets complex phenomena under micro units as an issue of order [17]. Exploring the interactions and qualitative changes in research topics in medical education according to the framework of complex systems theory will provide new answers regarding the knowledge network of medical education. Unlike previous quantitative studies on the issues in medical education, this paper aims to identify the phases of medical education distinguished by changing topics and explore the topics that emerge during the phases.

Therefore, the study utilizes SNA to investigate the interaction patterns among the issues in medical education by applying the framework of complex systems theory [18] and realistically contemplating the abstract knowledge network of medical education.

## Methods

In order to grasp the features of research trends in the field of medical education, the study extracted social-network keywords connected to terms from the title and abstract of available articles in Medline. Mesh terms were used during the process of retrieving articles from Pubmed, and the keyword extraction was conducted through text analysis. The information used for the analysis in this paper includes the title, abstract, and publication year of the paper. Since MeSH terms are not attached to all of the papers to be analyzed in this study, the authors extracted the keywords using the TextRank algorithm from the text consisting of the title and abstract. The TextRank algorithm is advantageous in that it provides high performance without being influenced by the linguistic characteristics of the text to be analyzed (Mihalcea R, Tarau P: TextRank - bringing order into texts. In: Proceedings of the Conference on Empirical Methods in Natural Language Processing, unpublished). A more detailed analysis was conducted by collecting the articles for analysis. This analysis included 1) category-setting through analysis of keyword similarity, 2) performing content analysis on the keywords, 3) analyzing the resulting network, and 4) conducting a trend analysis.

In June 2015, we searched PubMed for articles indexed under the “medical” major topic whose titles or abstracts included the term “medical education.” Our query terms included related terms such as “medical learner,” “medical teacher,” “medical teaching,” “medical training,” “medical learning,” and “medical education.” In this stage, two researchers reviewed and evaluated the list of keywords. For all of the papers, the extracted keywords with the use of the TextRank Algorithm underwent a refining process by two researchers. During the refining process, in order to refine the keywords, we looked at the whole list, checked and summarized the thesaurus, exception list, and defined words that needed refinement, and conducted a re-analysis of keywords. For instance, the research team deleted numbers or keywords such as “the”, “% +/”, “% <”, “% ci -0/3”, “(99 m) tc” which make it difficult to draw out the meaning of a keyword before data analysis. They also considered singular and plural keywords, such as “cardiac problem” and “cardiac problems,” as synonyms. Moreover, abbreviations were normalized by controlling them with a list of synonyms.

Then, as the first stage in the data analysis, the team generated a frequency matrix sized 3,030X53 that consisted of the yearly frequency of all the terms and the year of publication of each article. Next, to sort out the terms, the team calculated the weighted value of the terms by applying the Term Frequency–Inverse Document Frequency (TF–IDF) formula used in the field of information search [19]. The weighted value W_t,D_ was calculated using the formula below.

$$ {W}_{t,D}= normalized\  tf\left(t,D\right)\times \mathit{\log}\left(\frac{\left|Y\right|}{\left|\left\{y\in Y:\right.\left.t\  appears\ in\ y\right\}\right|}\right) $$

The tf(t,D) refers to the adjusted value of the sum of the frequencies of t of terms used in data collection using yearly frequency, and N from $\mathit{\log}\left(\frac{\left|Y\right|}{\left|\left\{y\in Y:\right.\left.t\  appears\ in\ y\right\}\right|}\right)$ refers to the yearly range 53 {y∈Y: t appears in y}.

TF–IDF is a weighted value used in text mining, and it indicates how many times a certain word appears in a given document. The higher the value of TF-IDF, the greater its importance; this also means that the word appears often. Therefore, the value multiplies DF (Document Frequency) with IDF (Inverse Document Frequency), a reciprocal number. Since this value increases with the frequency of a specific word and decreases with the number of documents containing the word out of the total number of documents, it filters the words that appear often in most documents [20].

In order to quantitatively calculate the relationship between MeSH terms, the research team calculated the Weighted Jaccard Similarity [20].

That is, the relationship between the terms and t was calculated with the formula below, using the yearly frequency information from the frequency matrix.

*Relatedness*(*S*, *T*) = $$ \frac{\sum_y mina\left({S}_y,{T}_y\right)}{\sum_y maxa\left({S}_y,{T}_y\right).} $$

Distributional Hypothesis [21] is the result of a study showing that when two words are used in the same context, these two words tend to have a similar meaning, and we assumed that there is a higher correlation between the two words if two keywords were used many times in the same year compared to the case where they were not. The science mapping principle dictates that the more related two elements are, the closer to each other they are positioned in a map [22]. This study based on the approach of distribution hypothesis and the science mapping principle for the correlation between words, the frequency value of each word’s annual appearance was used. The calculation method used in this study is a Weighted Jaccard similarity that used the appearance frequency of keywords. When using a Weighted Jaccard similarity, if two words are used together with high frequency in multiple years, they return a high similarity value.

Clustering of keywords was calculated using the Markov Cluster(MCL) Algorithm [23], which is widely applied to weight graphs in the computer science field, after constructing a graph with the keyword as the node of the graph and the similarity between the keywords as the weight of the edge between the nodes.

The MCL algorithm is a simple yet useful algorithm that is used for sequence data clustering in the biotechnology field which can be expressed as a weight graph. Therefore, it can be understood that the keywords with a high frequency of simultaneous appearance are used in the same context and have a higher correlation than other words in the same year. That is to say, they return a high similarity value.

### Data analysis and interpretation

In order to analyze the process of change in research topics in the medical education field, the study used the Frequency Matrix and the Weighted Jaccard Similarity and marked the times at which clear changes occurred, such as when new keywords rapidly emerged or diminished, using yearly similarities as cut-off points. The entire data collection process was separated into five phases based on the emergence of keywords, and each phase was analyzed using SNA. In order to conduct the network analysis, the input file for Gephi, a tool used in network analysis, was generated by calculating the relevance of terms for each phase using the methods mentioned above and representing the values as the relevance between nodes. The size of each node was expressed as the authority score obtained by the HITS (Hypertext Induced Topic Selection) algorithm of Gephi [24]. The authority score enabled the extraction of main research topics by using the mutual information between the nodes that comprised the network. Here, the authority score refers to the frequency of the reception of links [25].

## Results

The study used PubMed articles that were available for electronic search using MeSH terms in October 2015. From 1963, the year of the first publication related to medical education, to 2015, a total of 9,379 articles (with 162,866 keywords) on medical education were published in PubMed, with a slow increase over time and a rapid increase since the 2000s.

### Category-setting through analysis of keyword similarity

**Fig. 1:**
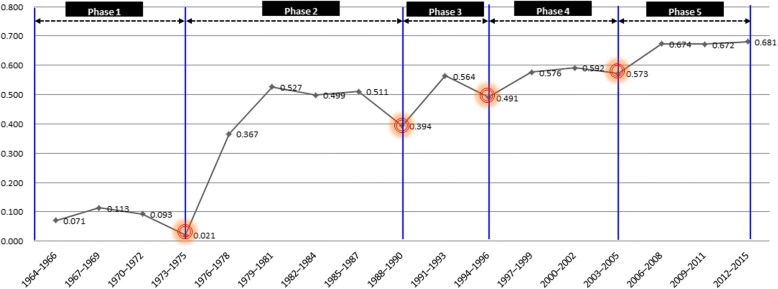
Phase-setting by similarity

Figure 1 shows the results of the analysis of keywords by year, arranged in three-year sections. In the graph, points at which similarity begins to increase after decreasing indicate a great increase in change in keywords; these were set as phase cut-off points.

On the basis of the similarity analysis by year, phase 1 was set to range from 1963 to 1975; phase 2 from 1976 to 1990; phase 3 from 1991 to 1996; phase 4 from 1997 to 2005; and phase 5 from 2006 to 2015. The next subsection characterizes these phases by keyword (and the keywords by phase).

### Content analysis in the key words

**Fig. 2:**
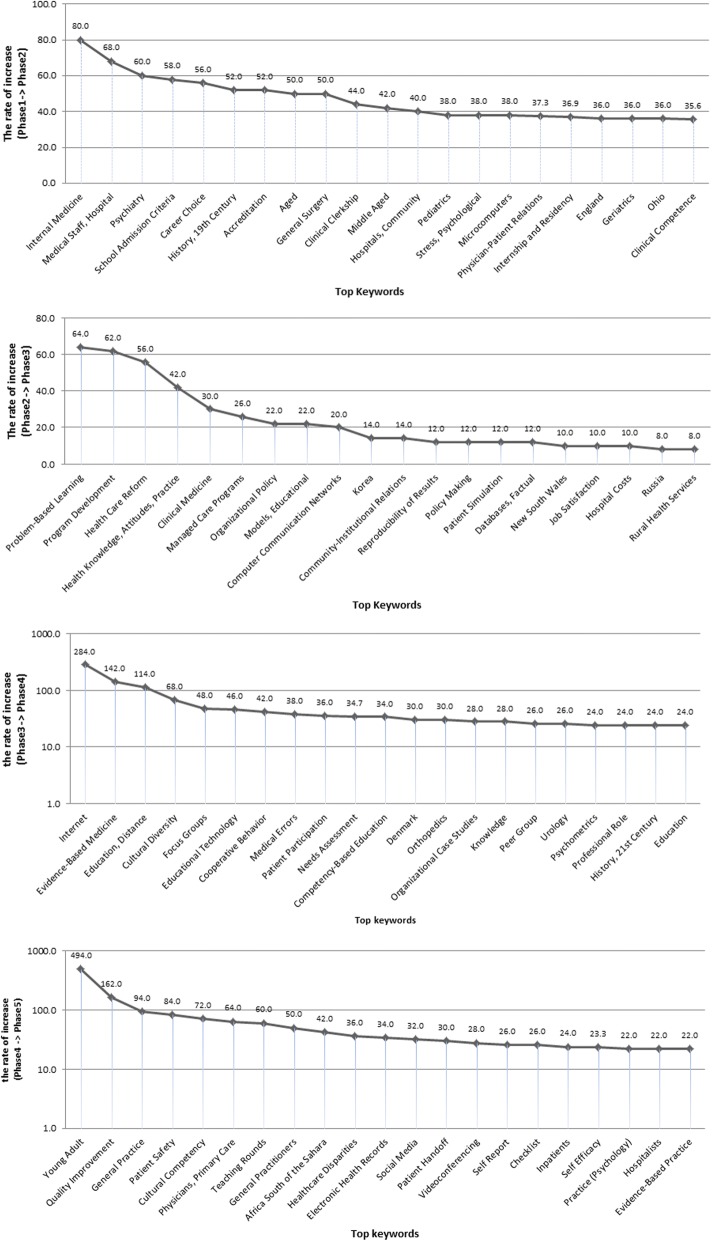
Top20 new keywords for each phase

Figure 2 shows increases and decreases in the top 20 keywords newly appearing in each phase. Keywords newly emerging as research topics were as follows for each phase: From phase 1 to 2, “Internship and Residency,” “Medical Staff, Hospital,” and “Psychiatry;” from phase 2 to 3, “Problem-Based Learning,” “Program Development,” and “Health Care Reform;” from phase 3 to 4, “Internet,” “Evidence-Based Medicine,” and “Education, Distance;” and from phase 4 to 5, “Young Adult,” “Quality Improvement,” “General Practice,” “Patient Safety,” “Cultural Competency,” and “Self-Efficacy.”

### Analysis of the resulting network

To systematically understand research trends and changes in knowledge structure in medical education over time, this study analyzed connections between keywords using social network analysis.

**Fig. 3:**
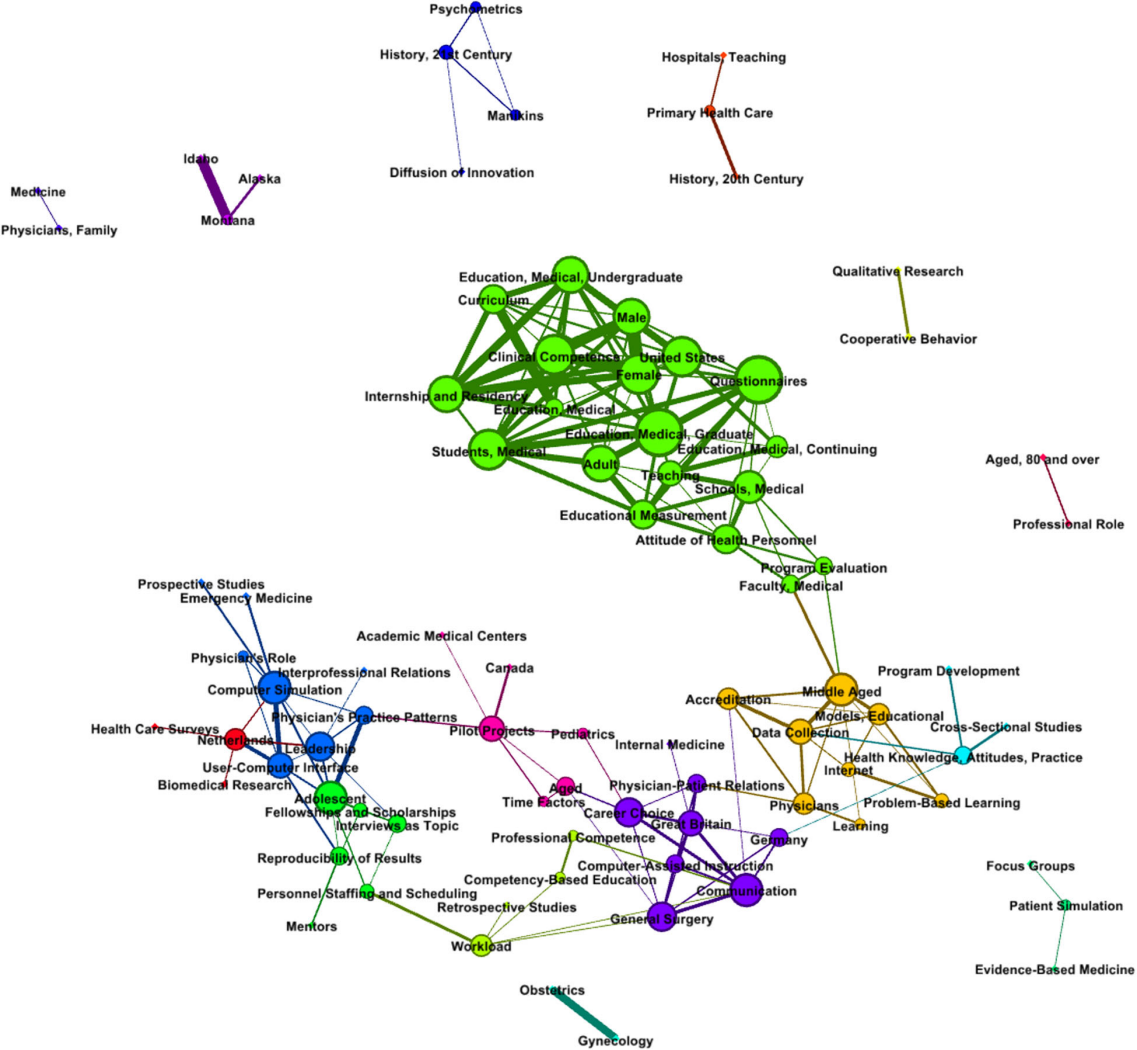
Topic clusters in medical education

Figure 3 shows a schematization of the network resulting from extraction of keywords with high connectivity and high weighted value for each phase. In all, 20 clusters were schematized.

Topics in cluster 1, the largest group (comprising 19 nodes), are as follows: “Education, Medical, Graduate,” “Questionnaires,” “Clinical Competence,” and “Internship and Residency.” Cluster 2 is made up of eight nodes, under the following topics: “Middle Aged,” “Data Collection,” “Accreditation,” and “Problem-Based Learning.” Cluster 3 has seven nodes, under the following topics: “Communication,” “Career Choice,” and “Computer-Assisted Instruction.” Topics in cluster 4 are gathered around “Computer Simulation,” “Leadership,” and “User–Computer Interface.” In cluster 5, “Competency-Based Education” and “Professional Competence” are the topics, and cluster 6 mainly deals with “Adolescents,” “Fellowships and Scholarships,” and “Interview as Topic.”

### Trend-watching: All five phases

**Fig. 4:**
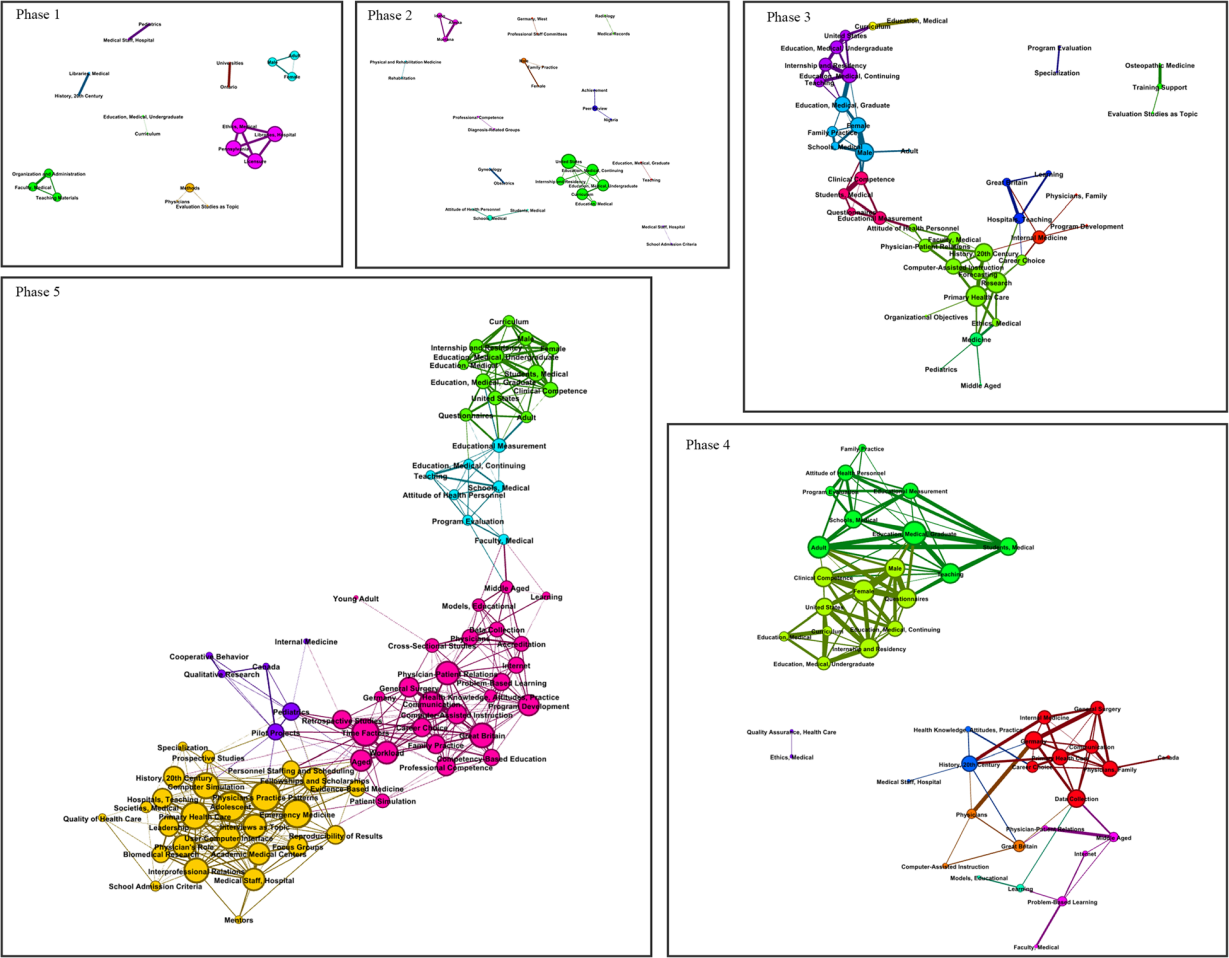
SNA of each historical phase of medical education

Figure 4 shows the SNA over all five phases, with detailed topic networks for each phase given in the Additional files 1, 2, 3, 4 and 5: Figures S1–S5. Phase 1 (1963–1975) showed lower connectedness among research topics compared to other phases, due to the difference in scale of article publication. Central keywords included “Education, Medical, Undergraduate,” “Curriculum,” “Male,” “Female,” and “Adult.” On the basis of these keywords, a subnetwork emerged, continuing up to phase 5. In phase 2 (1976–1990), connections between central keywords grew tighter, and new keywords appeared, including “Professional Competence,” “Attitude of Health Personnel,” and “Peer Review.” In phase 3 (1991–1996), connections among keywords such as “Clinical Competency,” “Educational Measurement,” and “Physician–Patient Relations” were enhanced. In phase 4 (1997–2005), the association of keywords with high connectedness became dual, and connections among keywords such as “Data Collection,” “Problem-Based Learning,” and “Health Knowledge, Attitudes, Practice” were enhanced. In phase 5 (2006–2015), the number and connectedness of keywords increased, and new keywords, such as “Computer-Assisted Instruction,” “Personal Staffing and Scheduling,” “User–Computer Interface,” “Professional Competency,” “Accreditation,” “Program Evaluation,” and “Educational Measurement,” appeared.

## Discussion

The study realistically contemplated the abstract knowledge network of medical education by identifying the network trends in medical education research topics through the use of SNA and investigating the use patterns in interactions by time. The study contains articles from the year when PubMed made it possible to electronically search for MeSH term in medical education articles, and coincidentally, this is consistent with Norman’s suggestion that a new generation of medical education has emerged [25].

The study identified five phases based on the changes in time indicated by clear differences in keyword similarities and identified newly derived keywords and networks in each phase. The clustering was conducted by deriving the keywords with high similarity using the appearance frequency values ​​of the two keywords and constructing a weighted graph based on the similarity between the keywords. This can be observed by clustering the sequences of the main keywords overall.

When the trend of the newly emerged keywords from five phases was analyzed, the keywords with the highest increase rates in all the phases, such as “Education,” “Medical,” “Humans,” “Curriculum,” “Continuing Medical Education,” and “Internship and Residency,” were similar to the keywords from a previous study by Lee K. [9]. Such repetition of research topics, as noted in the study by Eva K. W., probably occurs because studies in medical education are mostly observational [26].

In a similar study, Lee K. also analyzed the historical trends in medical publications in the field of medical education [9]. Even though the general trend appears similar, it did not distinguish the semantic unit that grasps the variations in the emergence of new keywords. Therefore, the study is meaningful in that it distinguishes between the phases by analyzing the interactions between medical education keywords using the complex systems framework.

Research in medical education has mostly been dominated by a positivist approach [26], and the emergence of new keywords with time represents the extent of the efforts being made to reflect social needs using the educational paradigm [27]. When the contents of topics that increased in a certain period or had newly emerged were analyzed, the first phase was characterized by important keywords being continuously mentioned in medical education, and new keywords such as “competency” or “accreditation” began to appear in the second phase. This could be due to the fact that the authentication program was formally declared in 1975 in order to improve the quality of medical education [28]. Considering that the top-cited articles in medical education began to contain reviews and research on competency since then, the studies seem to have been accumulated from this time [10]. Keywords that emerged in the third phase, such as “problem-based learning” and “computer communication networks” imply an increased interest in new education methods [4]. Keywords such as “competency-based assessments” and “outcome-based education,” which emerged in the fourth phase, represent the extension of research topics during the time period in which medical education became a topic of conversation [28]. Finally, the fifth phase is characterized by the emergence of keywords such as “quality improvement,” “patient safety,” “cultural competency,” and “self-efficacy,” which confirms that more research reflecting the trend is being conducted and realistic demands of medical education are being made. This period is also marked by increased interest in medical education and emphasis on the importance of evaluation, and thus, qualitative analysis and program evaluation were among the most important research topics (Fligstein N: Theoretical perspectives in medical education: past experience and future possibilities, unpublished). It appears that interest in quality improvement increased as social requirements for doctors gradually influenced educational institutions [27, 29, 30]. Therefore, various educational programs should be developed and evaluated with a focus on the effectiveness of medical education [31–34].

After exploring the keywords used in medical education research using SNA from a macro perspective, the research team analyzed the research trends of each phase by historical flow. When a network is considered to be one ecosystem, it corresponds with the principle of complex systems, and from the perspective of interpreting the flow of the network, the complex systems logic is presented as a new alternative [35]. Recently, there have been a number of discussions on the need to explore the nature of knowledge networks using complex systems theory [17].

The study was able to identify the process of improving the academic field in medical education by analyzing keywords in separate phases. This effort can be considered as a method of knowledge formation clearly distinguished from those used in previous studies. An emergency refers to a disorderly situation that arises as a result of complex network structures and patterns, and the system of such an emergency can be referred to as a complex system [18]. Unlike the analysis of keyword emergence, the flow of the phases studied by SNA is quite similar to the changing trend in human societies or networks [36]. This implies that the research topics in medical education resemble the emergence phenomena, as used in the complex phenomena. In other words, when examining the timely flow of keywords related to medical education, it can be noted that the newly emerged keywords form a network by interacting with each other. This, like the coevolution phenomena presented by complex systems theory, shows a similar phenomenon in which keywords evolve as they interact. As such, it seems like the trend in topics by phases derived through medical education keyword analysis is a part of the change process suggested by complex systems theory. At the same time, a cycle in which new research topics emerge, interact, and evolve should be formed [30, 37].

As the research team examined the research trends by phase, the features of each phase could be analyzed on the basis of complex systems theory: the waking phase (1963–1975), the birth phase (1976–1990), the growth phase (1991–1996), the maturity phase (1997–2005), and the expansion phase (2006–2015). And each name contained one the following meanings. The first period (1963–1975) is when keywords that served as central nodes for all the phases, such as “Education, Medical, Undergraduate” “Curriculum,” “Male,” “Female,” and “Adult,” appeared. This period forms the backbone of research in medical education, and shows the networks of basic levels. The second phase (1976–1990) is the period of the birth of medical education. The major keywords in the first phase focused on the subjects; however, the second phase is characterized by a focus on the keywords of properties, such as “Professional Competence,” “Attitudes of Health Personnel,” and “Peer Review.” In order for the subjects of medical education research to connect and be studied, it seems like the keywords representing the properties or characteristics should emerge and connect the subjects and foster the research. The third phase (1991–1996) was a period of growth for medical education, which is marked by the emergence of keywords in research methodology such as “Educational Measurement,” “Evaluation as Topic,” and “Questionnaire.” It appears that various methodologies have been tried in order to achieve qualitative improvement in subjects and properties. Ultimately, this seems to reflect the purpose of solving various problems in medical education, and it has been confirmed that suggested alternatives influenced and improved the academic field of medical education. The fourth phase (1997–2005) was a period of maturity, when keywords such as “Health Knowledge, Attitudes, and Practice,” “Ethics and Medicine,” and “Physician-Patient Relations” emerged. These keywords reflect an increasing interest in selecting physician candidates with high morality, with an emphasis on ethical responsibilities in medical education. At the same time, the trend represents the extension of research topics from analysis of general education to quality management [27]. From this perspective, various educational keywords in performance and competency are being connected in this phase. This trend could imply that the influence of general citizens’ requirements of physicians could have had effects on the education sector as well. The fifth phase (2006–2015) was a period of expansion, and major keywords such as “Computer Assisted Instruction,” “Personal Staffing and Scheduling,” “User-Computer Interface,” “Professional Competency,” “Accreditation,” and “Program Evaluation” emerged. Unlike other keywords, medical education keywords have larger network connectivity from the fifth phase, forming true network structures. While the first three phases are marked by the emergence of new keywords, the fourth phase is characterized by network formation. The fifth phase is called the period of expansion because networks are becoming highly concentrated and forming new networks.

However, the study has some limitations. First of all, since it is a quantitative study using SNA, it focused on terms related to medical education in Medline without taking into consideration the articles published in medical education journals such as JAMA(Journal of the American Medical Association), BMJ(British Medical Journal), JAMA internal medicine, etc. Thus, compared to the studies conducted by Rotgans JI, Wolf E Hautz et al., and Tutarel O, some articles are not reflected in this study [4, 11, 12]. Secondly, as in many other studies, the research team could only search for articles published in English. Thirdly, co-word analysis, co-citation analysis, and bibliographic coupling are among the most commonly used content analysis methods in the field of bibliometrics [38]. Co-word analysis used in this study is a method of analyzing a pattern in which a pair of terms (phrases) used in text in a specific field are analyzed at the same time to reveal the knowledge structure of the field. In this paper, the corelation between two words is used by frequency of use of words in common by year using the approach of distribution hypothesis. However, in order to better comply with the context-based distribution hypothesis, a new method is needed to calculate semantic relations between two words in the future. Fourthly, since this is a quantitative study, there is a need for interdisciplinary research focusing on issues in sociology, economics, or ecology. At the same time, future studies could focus on the establishment of new theories from current effectiveness verification studies. Lastly, a great amount of time and manpower was required for data collection, classification, and interpretation, because of which the study could not implement additional keyword analysis within the last one year. This calls for the development of a new research methodology that can readily analyze recent trends through SNA.

## Conclusions

As this shows, medical education research has focused not only on medical knowledge and practice (content) but also on research topics related to education theory as a social science (pedagogy) [31, 39]. Hence, for the development of medical education, a relevant community of work in related social science fields is also needed, and this work, from all disciplines, needs to be pursued in an integrated, interdisciplinary fashion, with fields and studies reflecting each other’s requirements and assumptions. In this way, a new and unique kind of medical education will develop, which will be crucial for the future of the field.

The study reinterpreted the changes in medical education using the complex systems theory, a mechanism in which various factors influence each other and collide into an order while forming a causal relationship. This confirmed that a unique legitimacy of medical education is being formed. Research in medical education is continuously improving and keeping pace with numerous social changes. Therefore, as educational and sociological theories integrate in the field of medicine, the medical education sector is expected to achieve independent development in the future.

## Additional files

Additional file 1: **Figure S1.** SNA of each historical phase1(1963–1975) of medical education (TIF 1645 kb)

Additional file 2: **Figure S2.** SNA of each historical phase2(1976–1990) of medical education (TIF 2932 kb)

Additional file 3: **Figure S3.** SNA of each historical phase3(1991–1996) of medical education. (TIF 3844 kb)

Additional file 4: **Figure S4.** SNA of each historical phase4(1997–2005) of medical education. (TIF 3575 kb)

Additional file 5: **Figure S5.** SNA of each historical phase5(2006–2015) of medical education. (TIF 2378 kb)

## Abbreviations

DF: Document Frequency
HITS: Hypertext Induced Topic Selection
IDF: Inverse Document Frequency
JAMA: Journal of the American Medical Association; BMJ: British Medical Journal
MCL: Markov Cluster
ME: medical education
SNA: Social Network Analysis
TF-IDF: Term Frequency–Inverse Document Frequency
