# Supplementary figures and images for: Research topics and trends in medical education by social network analysis
Source: BMC Med Educ. 2018 Sep 24;18:222. doi: 10.1186/s12909-018-1323-y (PMC6154904; doi:10.1186/s12909-018-1323-y)

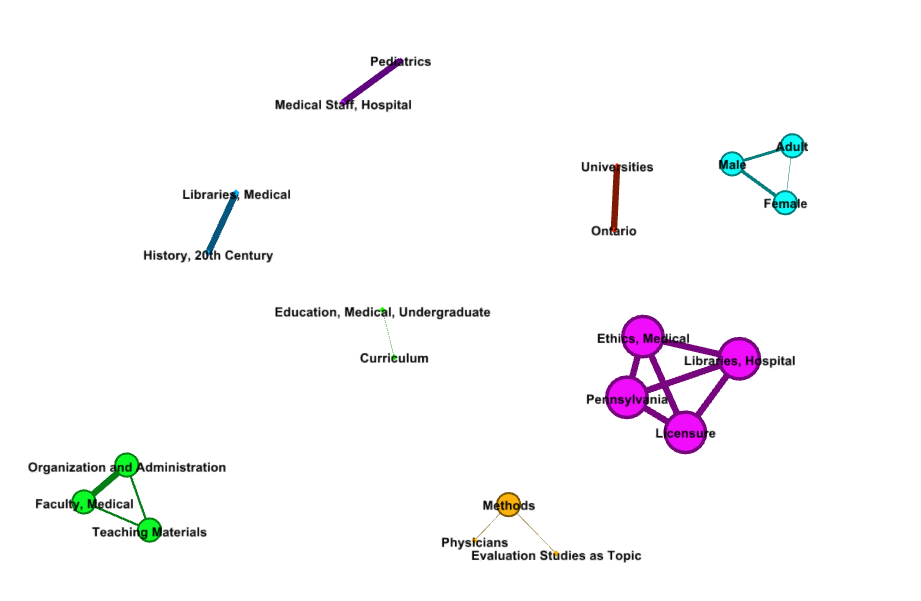

Supplement: Supplementary file 1 — Figure S1. SNA of each historical phase1(1963–1975) of medical education (TIF 1645 kb) [file 12909_2018_1323_MOESM1_ESM.tif]

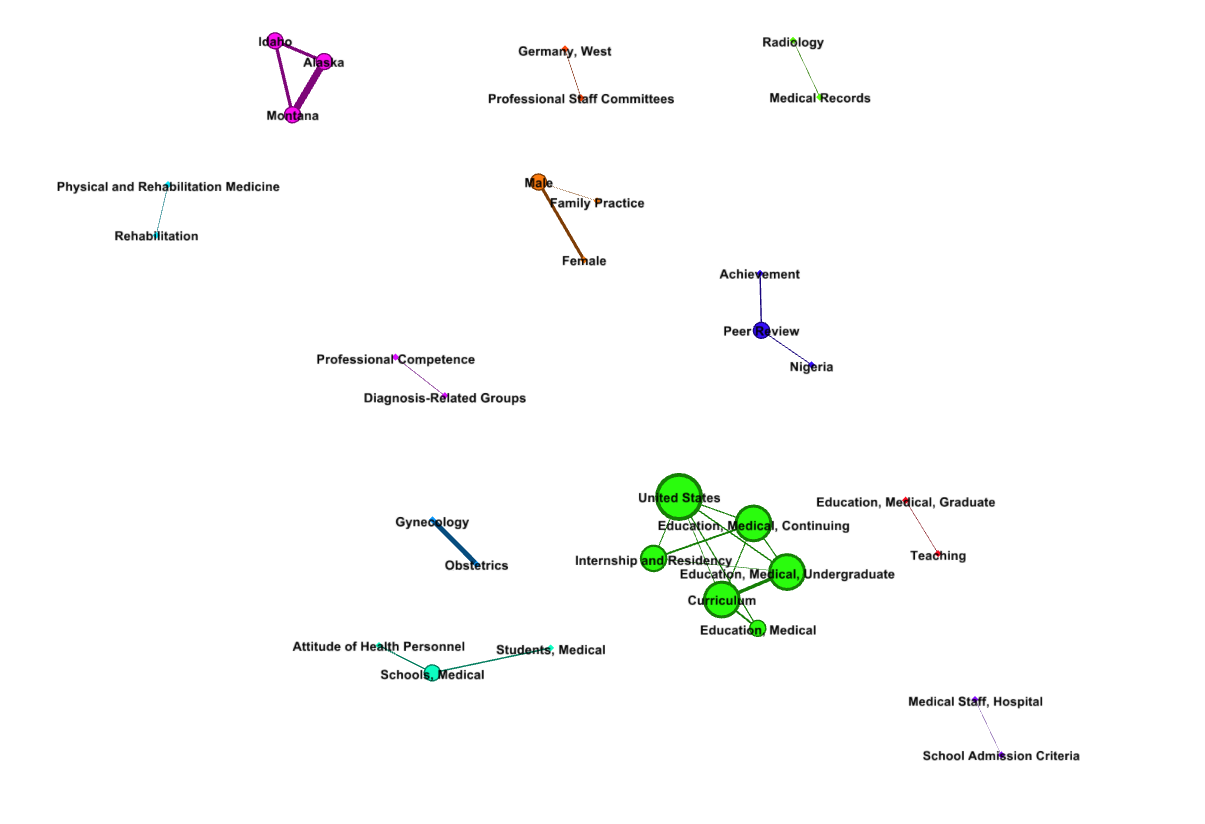

Supplement: Supplementary file 2 — Figure S2. SNA of each historical phase2(1976–1990) of medical education (TIF 2932 kb) [file 12909_2018_1323_MOESM2_ESM.tif]

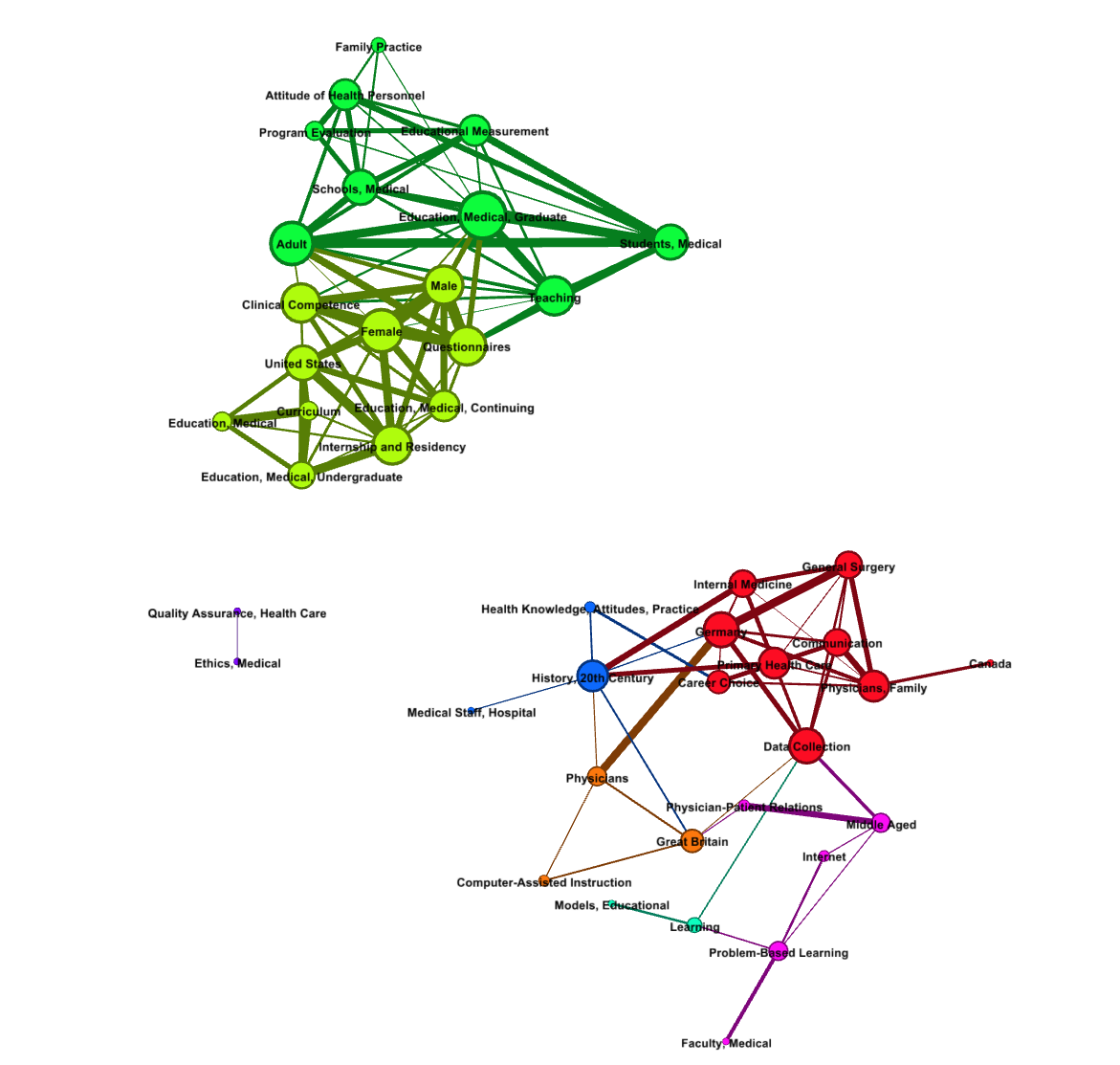

Supplement: Supplementary file 3 — Figure S3. SNA of each historical phase3(1991–1996) of medical education. (TIF 3844 kb) [file 12909_2018_1323_MOESM3_ESM.tif]

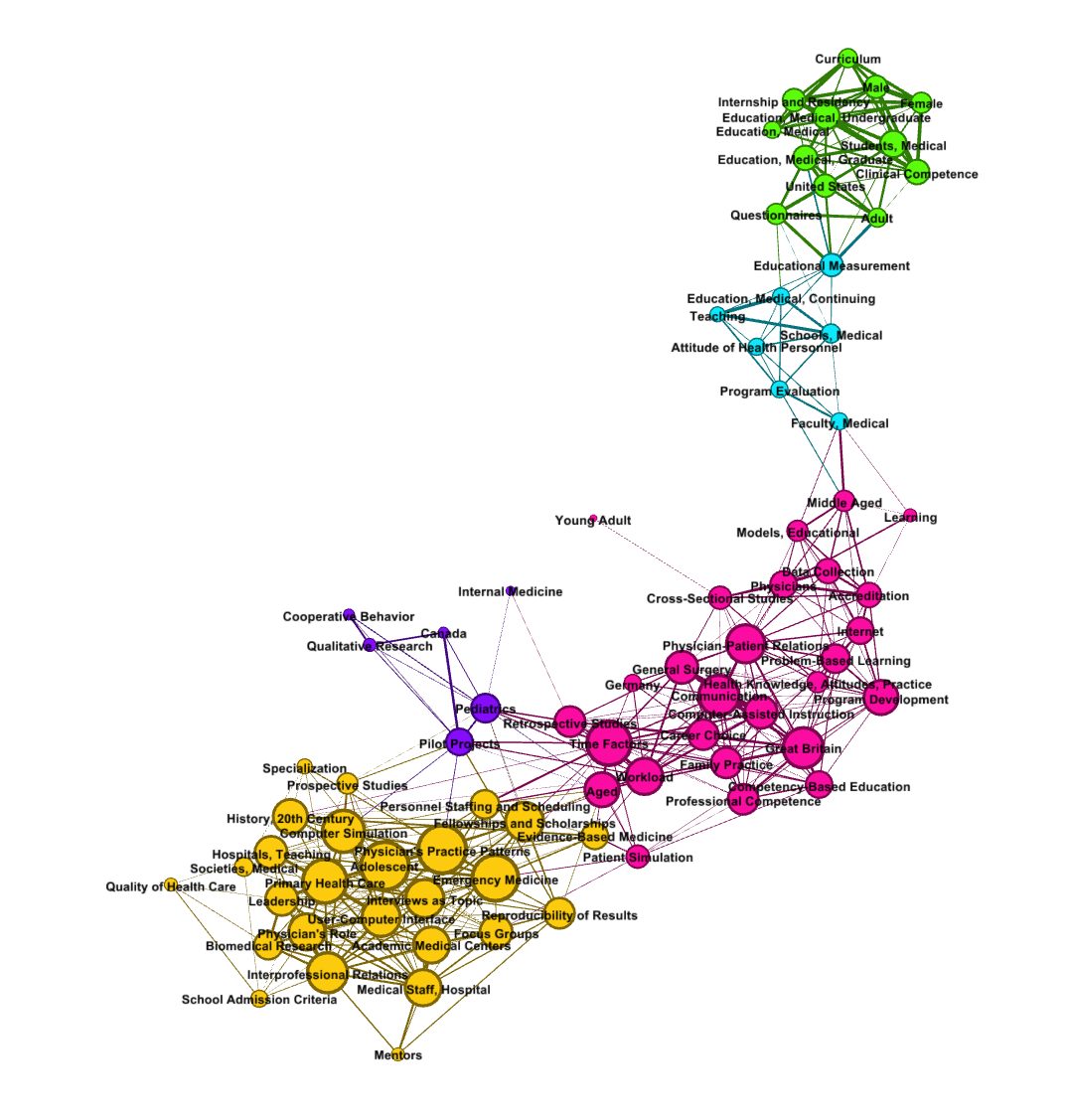

Supplement: Supplementary file 4 — Figure S4. SNA of each historical phase4(1997–2005) of medical education. (TIF 3575 kb) [file 12909_2018_1323_MOESM4_ESM.tif]

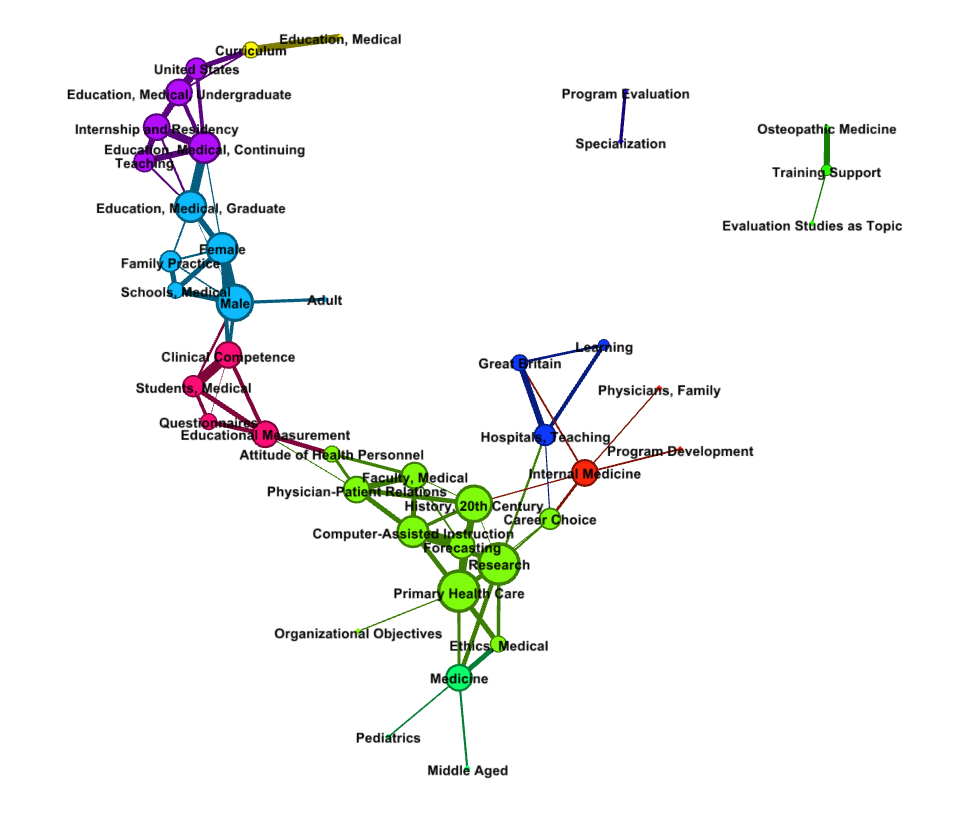

Supplement: Supplementary file 5 — Figure S5. SNA of each historical phase5(2006–2015) of medical education. (TIF 2378 kb) [file 12909_2018_1323_MOESM5_ESM.tif]
